# Belimumab in Systemic Lupus Erythematosus: From B-Cell Biology to Disease Modification

**DOI:** 10.3390/jcm15083173

**Published:** 2026-04-21

**Authors:** Marc Xipell, María Cecilia Garbarino, Cristina Serrano del Castillo, Laura Morantes, Carla Bastida, Ignasi Rodríguez-Pintó, Jose A. Gómez-Puerta, Gerard Espinosa, Luis F. Quintana, Ricard Cervera

**Affiliations:** 1 Department of Nephrology and Renal Transplantation, Hospital Clinic, 08036 Barcelona, Spain; 2 Reference Center for Complex Glomerular Diseases, Spanish Health System (CSUR), 08036 Barcelona, Spain; 3 Department of Medicine, University of Barcelona, 08036 Barcelona, Spain; 4 Institut d’Investigacions Biomèdiques August Pi I Sunyer (IDIBAPS), 08036 Barcelona, Spain; 5 Department of Autoimmune Diseases, Hospital Clinic, 08036 Barcelona, Spain; 6 Reference Centre for Systemic Autoimmune Diseases of the Catalan (XUEC), Spanish Health System (CSUR), 08036 Barcelona, Spain; 7 Department of Immunology, IIS-Fundación Jiménez Díaz University Hospital, 28040 Madrid, Spain; 8 Pharmacy Department, Hospital Clinic, 08036 Barcelona, Spain; 9 Department of Pharmacology, Toxicology and Therapeutical Chemistry, School of Pharmacy, University of Barcelona, 08007 Barcelona, Spain; 10 Department of Rheumatology, Hospital Clinic, 08036 Barcelona, Spain

**Keywords:** systemic lupus erythematosus, belimumab, BAFF inhibition, B-cell subsets, disease modification

## Abstract

Systemic lupus erythematosus (SLE) is a heterogeneous autoimmune disease in which B-cell dysregulation plays a central pathogenic role beyond autoantibody production. Advances in B-cell biology have led to the development of targeted therapies, including inhibition of the B-cell activating factor (BAFF) pathway. Belimumab, a monoclonal antibody that neutralizes soluble BAFF, modulates B-cell survival signals upstream, promoting progressive immunologic remodeling rather than rapid depletion. This review integrates current knowledge on BAFF-dependent B-cell biology with mechanistic, pharmacokinetic, and clinical data to provide a comprehensive framework for understanding belimumab’s effects in SLE and lupus nephritis (LN). Belimumab preferentially reduces transitional and naïve B cells, while memory B cells show a relative transient increase followed by a gradual return to baseline levels, reflecting redistribution rather than expansion, and long-lived plasma cells are largely unaffected. These effects result in progressive remodeling of B-cell compartment dynamics and contribute to broader modulation of adaptive immune amplification pathways. Pharmacokinetic data support a threshold-based model of BAFF neutralization, with exposure influenced by disease-related factors such as proteinuria in LN. Clinical response is primarily determined by baseline disease biology, with greater benefit observed in patients with serologically active disease and less established organ involvement. Across clinical trials and real-world studies, belimumab reduces disease activity and flares, enables glucocorticoid tapering, and slows organ damage accrual. In LN, it improves renal outcomes and reduces the risk of kidney-related events. Collectively, these findings support belimumab as a disease-modifying therapy in SLE. Further research is needed to refine patient selection and optimize treatment sequencing and combination strategies.

## 1. Introduction

Systemic lupus erythematosus (SLE) is a heterogeneous autoimmune disease in which B cells play a central pathogenic role beyond autoantibody production [1]. Advances in B-cell biology have shifted treatment strategies from broad immunosuppression toward targeted immunomodulation, with BAFF pathway inhibition emerging as a rational approach to restore peripheral tolerance and limit chronic immune activation [2].

Belimumab, the first biologic approved for SLE and later for lupus nephritis (LN), is supported by over a decade of clinical trial and real-world data [3,4,5]. These studies have defined a mechanism characterized by upstream modulation of B-cell survival signals, leading to gradual, biology-driven disease control rather than rapid depletion. However, questions remain regarding patient selection, predictors of response, and interpretation of outcomes in the context of disease heterogeneity and organ damage [6].

To inform this review, a structured literature search was conducted in PubMed/MEDLINE, complemented by manual screening of reference lists. Study selection was guided by relevance to the scope of the review, prioritizing pivotal clinical trials, key observational studies, and recent advances in belimumab and SLE/LN. This approach reflects a narrative, expert-driven review rather than a formal systematic review.

Here, we integrate current knowledge of BAFF-dependent B-cell biology with mechanistic, pharmacokinetic, and clinical data to provide a framework for understanding how belimumab modifies disease activity over time in SLE and LN.

## 2. Baff, B Cells, and Rationale for Targeting the Baff Pathway in SLE

### 2.1. Physiologic B-Cell Homeostasis and BAFF-Dependent Peripheral Tolerance

Human B-cell development begins in the bone marrow, where immature precursors give rise to transitional and subsequently mature naïve B cells that migrate to the circulation and secondary lymphoid organs [7]. Upon antigen encounter, B cells differentiate into antibody-secreting plasma cells—plasmablasts and plasma cells—or memory B cells. Beyond antibody production, B cells also contribute to immune regulation through antigen presentation, cytokine secretion, and integration of innate immune signals [8].

Plasma cells constitute the effector arm of humoral immunity. After antigen activation, a fraction of antibody-secreting plasmablasts—the immediate precursors of plasma cells—home to the bone marrow, where they occupy specialized survival niches that sustain long-term antibody production independently of ongoing antigen exposure [9]. Additional plasma cell populations reside in peripheral lymphoid tissues and inflamed organs, where they are typically short-lived but may include long-lived subsets in chronic inflammatory environments [10,11].

Memory B cells do not actively secrete antibodies but provide immunologic memory by mounting rapid secondary responses upon antigen re-exposure, mainly through differentiation into new plasma cells. Some may also re-enter germinal center reactions, generating newly refined memory B cells with further affinity maturation [12]. They circulate in blood and localize to secondary lymphoid organs and mucosal tissues, where they can be rapidly reactivated during inflammatory responses [13].

These processes are regulated by the TNF-family cytokines BAFF (B-cell activating factor) and APRIL (a proliferation-inducing ligand), produced mainly by myeloid antigen-presenting and stromal cells in response to inflammatory signals such as type I interferons and TNF-α [7,14,15]. BAFF is synthesized as a type II transmembrane protein that can be cleaved into a soluble form responsible for most systemic effects and acts as a key regulator of B-cell survival by setting the threshold for peripheral selection during early B-cell maturation [14].

**Figure 1:**
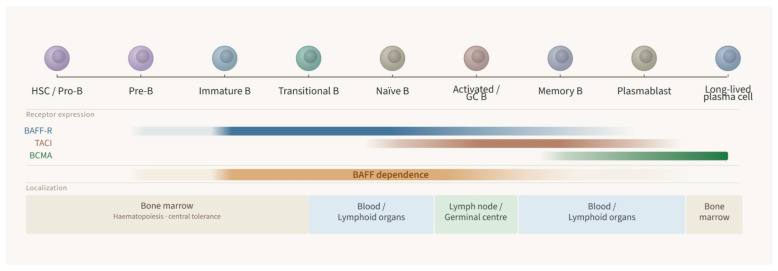
Stage-specific BAFF receptor expression and BAFF dependency throughout B-cell development.

BAFF and APRIL exert complementary roles across differentiated B-cell compartments [16]. Soluble BAFF signals through three receptors expressed on B lymphocytes—BAFF-R, TACI, and BCMA—whereas APRIL binds TACI and BCMA but not BAFF-R [17,18,19]. Their expression is developmentally regulated: BAFF-R predominates in transitional and naïve B cells, while TACI and BCMA are preferentially expressed on activated, memory, and antibody-secreting cells [19,20,21]. BAFF-R signaling is essential for B-cell survival and maturation and, in cooperation with B-cell receptor signaling, sets the threshold for peripheral selection of the naïve B-cell repertoire [22,23] (Figure 1).

TACI is expressed on activated and memory B cells and plasma cells, whereas BCMA is largely restricted to plasmablasts and long-lived plasma cells, and serves as a key survival receptor for antibody-secreting cells. Because plasma cells lack BAFF-R, their maintenance relies primarily on APRIL-driven signaling through BCMA (and to a lesser extent TACI), with partial redundancy from BAFF in sustaining plasma-cell survival niches [19,20].

Although memory B cells are largely independent of BAFF and APRIL for basal survival, BAFF can promote their activation and expansion under inflammatory conditions [23,24]. BAFF also supports immunoglobulin class-switch recombination and enhances responsiveness to T independent activation by innate immune signals, including Toll-like receptor pathways activated by nucleic acid–containing immune complexes. Together, BAFF and APRIL act as central regulators of B-cell homeostasis, integrating developmental cues, inflammatory signals, and peripheral tolerance mechanisms [16,25].

Importantly, because BAFF availability critically regulates the selection of transitional and naïve B cells and the survival of autoreactive clones in the periphery [23], increased BAFF levels can promote the persistence of potentially pathogenic B-cell populations and contribute to autoimmune disease, providing the biological rationale for therapeutic BAFF inhibition.

### 2.2. Disruption of B-Cell Homeostasis in SLE

In SLE, B-cell dysregulation arises from defective immune tolerance [26]. Defects in central and peripheral tolerance checkpoints—particularly at transitional and early mature B-cell stages—allow autoreactive B cells to enter the functional repertoire, where activation occurs within an antigen-driven inflammatory environment in which increased BAFF-mediated survival signals participate [27,28].

Nucleic acid sensing across innate and adaptive immune compartments further amplifies this dysregulated response. Cytokines produced by antigen-presenting cells, particularly type I interferons and TNF-α, promote naïve B-cell activation and germinal center reactions. At the same time, cell-free nucleic acids and immune complexes engage B-cell coreceptors [29], while endosomal Toll-like receptors—particularly TLR7 and TLR9—provide additional activation signals that promote B lymphocyte activation and plasmablast generation and expansion [30,31]. These innate immune pathways also induce BAFF production by myeloid antigen-presenting cells, including monocytes and dendritic cells, increasing BAFF availability and promoting the survival and expansion of autoreactive clones.

Multiple B-cell subsets contribute to autoantibody production in SLE [1,8]. Short-lived plasmablasts are a major source of dynamically regulated autoantibodies—including anti-dsDNA—whose titres fluctuate with disease activity and reflect sensitivity to upstream signals governing B-cell survival and differentiation. In contrast, long-lived plasma cells—which are relatively BAFF-independent and sustained through APRIL–BCMA signaling—produce more stable autoantibody responses, including anti-Sm, anti-Ro, and anti-La. Consequently, these antibody specificities are less reflective of current disease activity and less amenable to modulation by therapies targeting earlier B-cell stages [32,33].

Autoantibodies promote immune complex formation that engages Fc and complement receptors on innate and adaptive immune cells, driving inflammatory amplification loops and contributing to disease flares. In active SLE, antibody-secreting cells localize not only to the bone marrow but also to lymphoid tissues and inflamed organs, including the kidney in LN [34], where some may acquire long-lived features and sustain local autoantibody production [35].

Together, these immune alterations position BAFF as a central regulator of autoreactive B-cell survival and peripheral tolerance, linking innate immune activation to sustained humoral autoimmunity and providing a strong mechanistic rationale for therapeutic BAFF inhibition [2].

## 3. Mechanism of Action of Belimumab: Baff Neutralization and Modulation of B-Cell–Driven Disease Pathways

**Figure 2:**
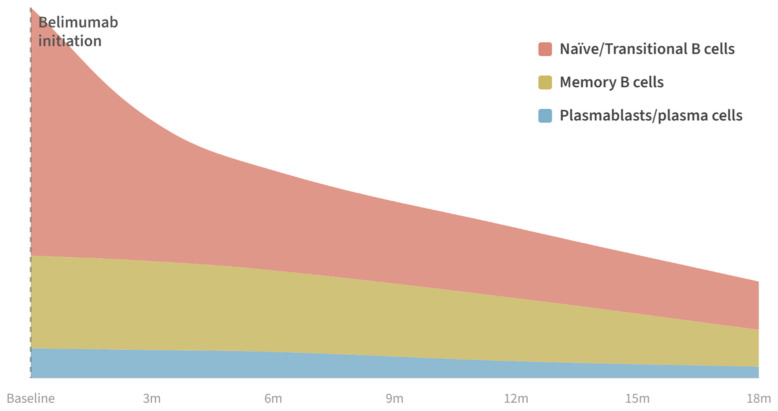
**Divergent kinetics of peripheral B-cell subsets during belimumab therapy in SLE.** Belimumab reshapes, rather than resets, the peripheral B-cell compartment. Proportional changes in circulating B-cell subpopulations over 18 months of belimumab therapy. Transitional/naïve B cells undergo early contraction, whereas the decline of memory B cells and plasmablasts is delayed. Data reflect peripheral blood only; individual patients with SLE may show considerable variability in baseline subset distribution and treatment response.

Belimumab is a fully human IgG1-λ monoclonal antibody that neutralizes soluble BAFF, preventing its interaction with BAFF receptors (BAFF-R, TACI, and BCMA) and thereby inhibiting BAFF-mediated B-cell survival signaling [36]. Mechanistically, it acts upstream by limiting BAFF-dependent survival signals, progressively reshaping peripheral B-cell selection while largely preserving pre-existing humoral immunity and long-lived plasma cells [22]. By reducing BAFF availability, belimumab increases competition at peripheral tolerance checkpoints, preferentially affecting early B-cell compartments and relatively sparing more differentiated populations. As a result, it induces gradual immunomodulation rather than rapid B-cell depletion, leading to sustained changes in B-cell subset distribution [37] (Figure 2).

### 3.1. Preferential Effects on Early B-Cell Compartments

BAFF inhibition primarily affects newly formed B cells [28]. Preclinical and clinical studies show marked reductions in transitional and naïve B-cell populations after belimumab exposure [36]. Circulating B-cell subsets may decline within weeks of treatment initiation, with sustained reductions over subsequent months, reflecting contraction of BAFF-dependent early compartments that can fall to a small fraction of baseline with continued therapy [22].

By limiting the generation of naïve B cells—including autoreactive clones—and their differentiation into short-lived plasmablasts and memory B cells, belimumab progressively reduces pathogenic B-cell output [22]. This upstream effect aligns with the delayed onset and gradual consolidation of clinical efficacy. Because belimumab neutralizes soluble BAFF without directly targeting B cells, it does not induce antibody-dependent cellular cytotoxicity [38]; consequently, CD20^+^ B-cell reductions on peripheral blood are slower and less pronounced than with B-cell–depleting therapies such as rituximab. Although belimumab can bind membrane-bound BAFF in experimental settings, this interaction appears to have limited relevance in vivo [38].

### 3.2. Memory B Cells: Redistribution and Delayed Remodeling

Memory B cells exhibit a more complex and delayed response to BAFF neutralization. In the first months of therapy, a transient relative increase in the proportion of circulating memory B cells is often observed [39,40]. With prolonged treatment, memory B-cell numbers gradually decline, predominantly affecting unmutated, germinal center-independent memory B cells (IgD+ CD27+ or IgD-CD27-), while class-switched IgG memory B cells (IgD-CD27+)—relevant for protective immunity, but also for potentially autoreactive memory cells —are relatively preserved [39,40,41,42].

Although some memory B cells are largely independent of BAFF for short-term survival, their naïve precursors generated in the bone marrow remain dependent on BAFF. Therefore, sustained inhibition of BAFF limits the replenishment of the memory compartment, contributing to its gradual decline. Consequently, belimumab does not directly deplete autoreactive memory B cells but instead progressively reshapes the memory B-cell compartment over time [40].

### 3.3. Plasma Cells, Immunoglobulins, and Autoantibody Specificity

Plasmablasts and plasma cells are differentially affected by BAFF inhibition. Short-lived circulating plasmablasts show modest reductions with sustained treatment, whereas long-lived plasma cells—particularly in the bone marrow—are largely preserved [43]. This reflects their dependence on APRIL–BCMA signaling, as well as other signals, which are not directly targeted by belimumab [21]. Consequently, observed changes primarily involve circulating and short-lived antibody-secreting cells rather than depletion of established plasma cell compartments. The decline in anti–double-stranded DNA antibodies during therapy likely reflects reduced generation of new autoreactive B cells, while pre-existing antibody production may persist due to the longevity of memory B cells and plasma cells [22].

Clinically, this translates into gradual and selective reductions in immunoglobulin levels. Total IgG typically declines modestly and remains within the normal range, without major safety concerns. Reductions are not uniform: pathogenic autoantibodies, particularly anti–double-stranded DNA IgG, tend to decrease more than total IgG, whereas protective antibody titers are largely preserved [40,44,45]. These findings support a mechanism in which belimumab limits the generation of new autoreactive plasmablasts rather than eliminating long-lived plasma cells responsible for protective immunity.

Autoreactive antibody-secreting cells may also persist in lymphoid tissues and inflamed organs, including the kidney in LN [46,47]. While short-lived plasmablasts within these compartments may be indirectly affected by BAFF inhibition, a minority may acquire long-lived features [48] and depend primarily on APRIL–BCMA signaling, thereby escaping direct targeting. Resolution of local inflammation may nonetheless indirectly destabilize these ectopic niches, potentially contributing to gradual reductions in autoantibody levels over time [49,50].

### 3.4. Modulation of Immune Amplification Pathways and Translational Implications

Beyond quantitative effects on B-cell subsets, belimumab attenuates immune amplification pathways central to SLE pathogenesis. By reducing autoreactive B cells and immune complex formation, it reduces plasmacytoid dendritic cell activation and downstream type I interferon production. Given that interferon signaling upregulates BAFF expression, BAFF neutralization may disrupt this pathogenic feedback loop linking innate and adaptive immunity. In addition, BAFF enhances B-cell responsiveness to B-cell receptor and Toll-like receptor co-signaling, including in partially T-independent activation contexts, thereby promoting inflammatory differentiation and cytokine production. Accordingly, its inhibition exerts broader immunomodulatory effects beyond direct changes in B-cell numbers [16,22].

Clinically, belimumab is best understood as a therapy that progressively modulates B-cell biology rather than inducing rapid depletion. This profile aligns with delayed onset of efficacy, preferential benefit in serologically active disease, sustained reductions in anti–double-stranded DNA antibodies, complement normalization, and gradual reductions in flare frequency [51,52]. The preservation of humoral immune memory—driven primarily by somatically mutated, class-switched memory B cells and long-lived plasma cells—likely accounts for its favorable safety profile compared with more intensive B-cell–depleting approaches [45,53,54,55].

Overall, belimumab reshapes peripheral B-cell selection, limits autoreactive B-cell output, and attenuates immune amplification loops driving chronic inflammation. Its limited effects on tissue-resident plasma cells and established humoral memory suggest greater efficacy when introduced before autoreactive immunity becomes deeply established within protected survival niches [56,57].

## 4. Pharmacokinetics and Pharmacodynamics of Belimumab: Implications for Clinical Response

Belimumab exhibits pharmacokinetic properties typical of an IgG1 monoclonal antibody, including low systemic clearance, a small volume of distribution, and a long half-life [58,59]. It displays linear pharmacokinetics and first-order elimination across doses of 1–20 mg/kg, without clinically relevant target-mediated drug disposition at doses ≥1 mg/kg, indicating that target binding does not meaningfully influence pharmacokinetics within the therapeutic range. It is primarily eliminated through intracellular proteolytic catabolism within the reticuloendothelial system, and its large molecular size (~147 kDa) precludes glomerular filtration, rendering renal clearance negligible. Accordingly, reduced kidney function has minimal impact on drug exposure, supporting its use without routine dose adjustment across varying degrees of renal function in SLE and LN [60].

In population pharmacokinetic models, body size is the main determinant of belimumab clearance, justifying the weight-based intravenous dosing (10 mg/kg). The fixed subcutaneous regimen of 200 mg weekly is supported by PK analyses showing that body size–related variability in exposure remains within clinically acceptable limits. Baseline proteinuria correlates positively with clearance, likely due to increased non-specific protein catabolism, greater transcapillary escape and urinary losses from glomerular barrier disruption [61,62]. Ethnicity, age, sex, concomitant immunosuppressants, and disease activity do not have clinically meaningful effects on belimumab exposure after accounting for body size [63,64].

Following intravenous administration, belimumab undergoes a rapid distribution phase (1–2 days) followed by a slower elimination phase, with a terminal half-life of approximately two weeks. Systemic clearance is ~4–7 mL/day/kg and the steady-state volume of distribution ~3.5–5 L, consistent with distribution largely confined to vascular and interstitial spaces and FcRn-mediated recycling [63,65,66]. Following subcutaneous administration, belimumab is absorbed via lymphatic transport, reaching peak concentrations after several days with a bioavailability of ~75–80% [58]. Overall exposure, clearance, and half-life are comparable between intravenous and subcutaneous formulations, supporting pharmacokinetic bridging and similar clinical efficacy across dosing regimens [58,67,68,69].

Belimumab also exhibits low immunogenicity, although its assessment is partially limited by drug interference in anti-drug antibody assays. In phase III SLE trials, persistent anti-drug antibodies were infrequent (0.7–4.8%) and not associated with safety signals or clinically meaningful effects. Similar findings have been reported with the subcutaneous formulation. Overall, immunogenicity does not appear to meaningfully impact belimumab pharmacokinetics, safety, or efficacy [51,52,62,70,71,72,73].

### 4.1. Exposure–Response Relationships and Dose Considerations

During clinical development, belimumab was evaluated across a wide intravenous dose range (1–20 mg/kg), enabling assessment of exposure–response relationships [66,74]. No consistent dose-dependent gradient was observed for key serological biomarkers, including complement levels and anti–double-stranded DNA titers [74]. In the phase III BLISS-52 and BLISS-76 trials, both the 1 mg/kg and 10 mg/kg intravenous regimens increased SRI response rates versus placebo at week 52, with both doses meeting the primary endpoint in BLISS-52 and only the 10 mg/kg dose achieving statistical significance in BLISS-76. In BLISS-76, SRI response rates at week 76 remained numerically higher with belimumab than with placebo but did not reach statistical significance, and differences between the two active doses were generally modest across secondary endpoints [75,76,77].

These findings support a threshold-based pharmacodynamic model, whereby BAFF neutralization is largely achieved once sufficient exposure is reached, rather than increasing proportionally with dose. Differences between dosing regimens likely reflect how consistently individual patients achieve this threshold rather than graded increases in biological effect [78].

### 4.2. Proteinuria, Exposure, and Clearance

Underlying damage to the glomerular filtration barrier, reflected by proteinuria, is a clinically relevant determinant of belimumab exposure, particularly in LN [79]. Disruption of the filtration barrier may increase the urinary loss of large proteins and enhance clearance of monoclonal antibodies, leading to reduced systemic exposure in patients with high-grade proteinuria [80]. Population pharmacokinetic analyses from BLISS-LN showed that higher baseline proteinuria is associated with increased belimumab clearance, with early exposure up to ~50% lower in patients with nephrotic-range proteinuria [65,68].

However, as renal inflammation improves and proteinuria declines, belimumab clearance approaches values seen in patients with low or absent proteinuria, reducing between-patient pharmacokinetic variability and stabilizing exposure over time [68]. This suggests that reduced early exposure largely reflects disease-related factors that may partially normalize with treatment. Despite this, exposure–response analyses stratified by proteinuria have not shown consistent efficacy gains with higher exposure. These findings do not support routine dose escalation beyond the approved regimen in patients with high proteinuria outside clinical trials.

### 4.3. Clinical Predictors of Response

Clinical response to belimumab is largely determined by baseline disease biology, particularly the degree to which autoreactive responses remain BAFF-dependent [60,78]. From a mechanistic perspective, earlier disease stages—when autoreactive responses are driven predominantly by plasmablasts and short-lived plasma cells—are more amenable to BAFF inhibition, before long-lived plasma cell compartments become established. Accordingly, patients with serologically active SLE and less established disease —likely reflecting earlier, more BAFF-dependent immune responses—consistently derive greater benefit [6].

In LN, baseline proteinuria has both pharmacokinetic and prognostic implications: patients with lower levels achieve renal response more rapidly and more frequently, whereas those with nephrotic-range proteinuria tend to respond more slowly and less often [60,81]. Importantly, a lower probability of formal response in patients with high proteinuria does not imply absence of clinical benefit. Post hoc analyses show that belimumab reduces the risk of kidney-related events or death in this population despite lower rates of complete response by composite endpoints. This underscores that proteinuria reflects both disease severity and pharmacokinetic factors, and that meaningful benefit may extend beyond dichotomous response definitions, particularly in advanced or highly active disease [72].

Beyond baseline characteristics, early on-treatment biological changes may further refine response prediction. Post hoc analyses of the BLISS trials indicate that greater early reductions in naïve and transitional B cells at weeks 12 and 52, and reduction of plasmablasts at week 52, are associated with protection against renal flares, while a rapid decline followed by rebound in short-lived plasma cells and plasmablasts may identify patients at higher risk of flare [82,83].

## 5. Clinical Implications and Disease Modification with Belimumab in Systemic Lupus Erythematosus

### 5.1. Long-Term Immunologic Effects and Disease Trajectory

Beyond quantitative changes in B-cell subsets, longitudinal studies indicate that belimumab induces broader immunologic reprogramming. Sustained treatment attenuates type I interferon—and BAFF-related gene signatures and partially normalizes disease-associated transcriptional pathways [84], alongside progressive remodeling of the B-cell compartment with reductions in transitional and naïve B cells and decreased plasmablast output [22].

These changes support the concept that BAFF neutralization recalibrates upstream immune circuits rather than simply depleting B cells, providing a biological basis for the delayed onset of response and progressive stabilization of disease activity with long-term treatment.

### 5.2. Effects on Disease Activity, Flares, and Organ Damage Accrual

Belimumab reduces disease activity and flares across the clinical spectrum of SLE, as demonstrated in phase III trials and real-world studies. In BLISS-52 and BLISS-76, add-on belimumab improved SRI-4 response rates at 52 weeks (58% and 43.2%, respectively) [51,70] (Table 1). These effects are mirrored in real-world cohorts, with high rates of LLDAS and DORIS remission [85,86], enabling glucocorticoid tapering and contributing to reduced organ damage accrual. Long-term data show that 85.1% of patients have no progression in SLICC Damage Index over 5–6 years [87].

In LN, BLISS-LN demonstrated higher renal response and complete remission rates with belimumab plus standard therapy, along with reduced risk of kidney-related events or death [88]. Post hoc analyses showed a 55% reduction in LN flare risk and slower eGFR decline, including fewer sustained 30–40% reductions in kidney function [89].

Clinical benefit is greatest in patients with high baseline disease activity (SELENA-SLEDAI ≥ 10) and serological activity (anti–double-stranded DNA positivity and hypocomplementemia) [51,70,86]. Patients with musculoskeletal and mucocutaneous involvement also respond well [90], while in LN, higher response rates are observed in proliferative forms with baseline proteinuria <3 g/g [89].

Early intervention also appears to improve outcomes. Patients with shorter disease duration (≤2 years) or newly diagnosed SLE achieve remission more rapidly and experience greater glucocorticoid-sparing effects. Similarly, absence of baseline organ damage (SDI = 0) is an independent predictor of clinical response and sustained low disease activity [86,91]. Economic analyses further indicate that early use of belimumab improves health outcomes, reduces medical costs, and facilitates sustained glucocorticoid tapering or discontinuation, supporting its integration into treat-to-target strategies for SLE [92,93].

The concept of disease-modifying therapy (DMT) in SLE has been defined as a treatment that achieves sustained minimization of disease activity with the fewest treatment-associated toxicities, while slowing or preventing the accrual of irreversible organ damage (including, in LN, progression to end-stage kidney disease), typically accompanied by substantial reduction or discontinuation of glucocorticoid treatment [94]. Subsequent studies evaluating SLE therapies that fulfil these criteria have identified belimumab as a drug that meets DMT standards for extrarenal manifestations [95] and for LN [96]. Overall, by reducing disease activity and flares, limiting corticosteroid exposure, and slowing organ damage accrual, belimumab can be considered a DMT in SLE [97].

### 5.3. Safety Profile and Clinical Considerations for Treatment Monitoring

Belimumab has demonstrated a generally favourable long-term safety profile [98,99], with rates of serious adverse events and serious infections comparable to those observed with standard therapy alone, and long-term extension studies reporting maintained safety and efficacy for up to 13 years of exposure [100]. Serious infections most commonly involve pneumonia, urinary tract infections, skin and soft-tissue infections, and bronchitis, while herpes zoster and rare opportunistic infections have been reported, warranting vigilance in patients with substantial immunosuppression or relevant comorbidities [101]. The phase 4 BASE trial, specifically designed to assess adverse events of special interest, reported comparable rates of serious infections and malignancies between belimumab and placebo, while documenting a higher incidence of serious depression and suicide-related events in the belimumab arm, reinforcing the importance of routine psychiatric screening before and during treatment, despite the low absolute incidence of these events [102]. From a practical standpoint, monitoring requirements are similar to those of other biologic therapies in SLE, including periodic assessment of disease activity, complete blood count, renal and liver function, immunoglobulin levels, and serological markers. Vaccination status should be optimized prior to treatment initiation, live vaccines avoided during therapy, and ongoing treatment reconsidered in the setting of serious infections or sustained hypogammaglobulinaemia.

## 6. Conclusions

Belimumab is a targeted therapy for SLE that modulates B-cell–driven immune dysregulation through BAFF neutralization. By acting upstream, it reshapes peripheral B-cell selection, primarily reducing transitional and naïve B cells while largely preserving memory immunity, mainly somatically mutated and switched memory B cells and long-lived plasma cells. Clinically, this translates into gradual but sustained reductions in disease activity and flares, meaningful glucocorticoid sparing, and improved renal outcomes in LN [89,103,104]. The delayed onset of benefit reflects progressive immunologic remodeling rather than rapid immunosuppression, supporting a disease-modifying effect.

Patients with serologically active disease—likely reflecting greater dependence on BAFF-driven pathways—derive the greatest benefit. Compared with more intensive B-cell–depleting strategies, belimumab preserves protective immunity, contributing to its favorable safety profile. Future studies should refine patient selection and define optimal sequencing and combination strategies with other biologic therapies.

## Figures and Tables

**Table 1:** Clinical trials of belimumab in Systemic Lupus Erythematosus and Lupus Nephritis. AESI: Adverse Events of Special Interest; ANA: antinuclear antibodies; anti-dsDNA: anti-double stranded DNA antibodies; CI: confidence interval; IV: intravenous; LN: lupus nephritis; PERR: Primary Efficacy Renal Response; SC: subcutaneous; SELENA-SLEDAI: Safety of Estrogens in Lupus Erythematosus National Assessment–SLE Disease Activity Index; SLE: systemic lupus erythematosus; SLEDAI-2K: Systemic Lupus Erythematosus Disease Activity Index 2000; SOC: standard of care; SRI-4: SLE Responder Index-4; SRI-SLEDAI-2K: SLE Responder Index using SLEDAI-2K.

Trial	Phase	Population	Primary Endpoint	Treatment Arms	Results	Year of Publication
**BLISS-52**	Phase III	Active SLE (SELENA-SLEDAI ≥ 6 and ANA and/or anti-dsDNA positive); n: 867; Latin America, Asia-Pacific, Eastern Europe	SRI-4 at Week 52	Belimumab 1 mg/kg + SOC; Belimumab 10 mg/kg + SOC vs. Placebo + SOC	Primary endpoint met: 51% 1 mg/kg (*p* = 0.01); 58% 10 mg/kg (*p* = 0.0006) vs. 44% placebo	2011
**BLISS-76**	Phase III	Active SLE (SELENA-SLEDAI ≥ 6 and ANA and/or anti-dsDNA positive); n: 819; North America and Europe	SRI-4 at Week 52	Belimumab 1 mg/kg + SOC; Belimumab 10 mg/kg + SOC vs. Placebo + SOC	Primary endpoint met for 10 mg/kg: 43% vs. 33.5% placebo (*p* = 0.017); 1 mg/kg: 40.6% (*p* = 0.089)	2011
**BLISS-SC**	Phase III	Active SLE (SELENA-SLEDAI ≥ 8 and ANA and/or anti-dsDNA positive); n: 836; multinational	SRI-4 at Week 52	Belimumab 200 mg SC weekly + SOC vs. Placebo + SOC	Primary endpoint met: 61.4% vs. 48.4% placebo (*p* = 0.0006); hypocomplementemic patients: 64.6% vs. 47.2% placebo (*p* = 0.001)	2017
**BLISS-NEA**	Phase III	Active SLE (SELENA-SLEDAI ≥ 8 and ANA positive); n: 677; Northeast Asia (China, Japan, South Korea)	SRI-4 at Week 52	Belimumab 10 mg/kg IV + SOC vs. Placebo + SOC	Primary endpoint met: 53.8% vs. 40.1% placebo (*p* < 0.001)	2018
**BLISS-LN**	Phase III	Active LN (biopsy-proven class III, IV, or V LN with proteinuria requiring induction therapy); n: 448; multinational	PERR at Week 104	Belimumab 10 mg/kg IV + SOC vs. Placebo + SOC	Primary endpoint met: 43% vs. 32% placebo (*p* = 0.03)	2020
**EMBRACE**	Phase III–IV	Active SLE (SELENA-SLEDAI ≥ 8 and ANA and/or anti-dsDNA positive); n: 448; patients of Black/African ancestry	SRI-SLEDAI-2K at Week 52	Belimumab 10 mg/kg IV + SOC vs. Placebo + SOC	Primary endpoint not met: 48.7% vs. 41.6% placebo (*p* = 0.106)	2022
**BASE**	Phase IV	Active SLE (ANA and/or anti-dsDNA positive); n: 4018; multinational	All-cause mortality and AESIs up to 52 weeks	Belimumab 10 mg/kg IV + SOC vs. Placebo + SOC	Primary endpoints: No excess mortality or major AESIs vs. placebo; higher rates of serious depression and suicidality with belimumab	2024

## Data Availability

No new data were generated or analyzed in support of this review.
